# The potential role of incretin therapy in the hospital setting

**DOI:** 10.1186/s40842-015-0005-5

**Published:** 2015-07-01

**Authors:** Jennifer J. Macdonald, Shristi Neupane, Roma Y. Gianchandani

**Affiliations:** 1 Division of Internal Medicine, University of Michigan Medical Center, 1500 E. Medical Center Drive, 3116Q Taubman Center, SPC 5368, Ann Arbor, MI 48109 USA; 2 Division of Metabolism, Endocrinology & Diabetes, University of Michigan Medical Center, Domino’s Farms, Lobby G, Suite 1500, 24 Frank Lloyd Wright Drive, P.O. Box 482, Ann Arbor, MI 48106-0482 USA

**Keywords:** Hyperglycemia, Glucagon-like peptide-1 receptor agonist, Dipeptidyl peptidase-4 inhibitor, Inpatient, Incretin

## Abstract

Hyperglycemia has been associated with increased morbidity and mortality in hospitalized patients. Insulin has traditionally been the drug of choice for managing hyperglycemia in this setting, but carries a significant risk of hypoglycemia. Incretin-based therapies, including glucagon-like peptide-1, glucagon-like peptide-1 receptor agonists and dipeptidyl peptidase-4 inhibitors, have potential use in the hospital. These agents have a relatively low risk of hypoglycemia, favorable short-term side effect profile, and can be used alone or in combination with insulin. Several small studies have supported the safety and efficacy of incretin therapies in the inpatient setting with the majority of data coming from the intensive care setting. Large-scale clinical studies are needed to further evaluate the potential role of incretins in the management of inpatient hyperglycemia.

## Introduction

Hyperglycemia in the inpatient setting is commonly encountered in patients with and without known diabetes. While patients with diabetes account for an estimated 25-30 % of adult admissions, a significant percentage of hospitalized hyperglycemic patients have either unrecognized diabetes or stress hyperglycemia [1–3]. Hyperglycemia is now a well-established predictor of morbidity and mortality in patients with both diabetes and stress hyperglycemia [4]. Several randomized controlled trials evaluating tight glucose control (BG goal of 80–110 mg/dl) in the hospital have had conflicting outcomes, therefore treatment targets of blood glucose are more liberal now (140–180 mg/dl0) [5]. Tight glucose targets may be beneficial in select patient populations such as post-operative, steroid-induced, or trauma-related hyperglycemia [6, 7], but this is limited by hypoglycemia, which is also a known predictor of poor outcomes [7, 8]. Given the increasing prevalence of hyperglycemia in the hospital, there is great interest in providing effective glycemic management while avoiding hypoglycemia.

A wealth of clinical experience using insulin in the inpatient setting exists, where it remains the preferred method of glycemic control [5, 9]. Insulin given intravenously has several advantages including a short half-life and the ability to be rapidly titrated. However, insulin therapy is labor intensive and requires extensive training both for intravenous and subcutaneous use. Additionally, meal dosing is challenging in the hospital and needs to be timed appropriately with food and titrated to the percentage of meal consumed. Hypoglycemia remains a common problem with insulin and has an estimated prevalence of 12 % even in academic medical centers [10]. Insulin has been identified as one of the most common causes of preventable adverse drug reactions [11, 12]. Although insulin therapy has been shown to improve select outcomes [6], it fails to consistently reduce mortality in hospitalized hyperglycemic patients [13]. Given these drawbacks, identification of other glucose lowering agents that are safe, effective, and less labor intensive than insulin would be of great clinical utility in the inpatient setting.

Among the hypoglycemic agents introduced within the last decade, incretins have shown the most promise for inpatient use. They carry a low risk of hypoglycemia, which is particularly important in the inpatient setting where changes in oral intake, insulin timing, and comorbid conditions such as acute kidney injury can put patients at risk [14]. Additionally these drugs are already well established in the outpatient setting [15, 16]. Here we review the data for the use of incretin-based therapies both in the intensive care unit (ICU) and general ward settings.

## Review

### Physiology of incretins

The incretin effect is the observation that an enteral glucose load raises insulin levels more than an equivalent intravenous glucose load [17]. The 2 major incretins in the human body are glucose-dependent insulinotropic polypeptide (GIP) and glucagon-like peptide-1 (GLP-1) [18], which are released in response to glucose and fat ingestion by the K-cells and L-cells of the intestines respectively [18, 19]. Incretins have wide ranging metabolic effects which include increased insulin secretion from pancreatic beta cells, decreased glucagon secretion, decreased glucose production in the liver, increased glucose uptake in the muscle, slowed gastric emptying, and decreased appetite [18–20]. The suppression of glucagon by GLP-1 declines towards normoglycemia, as does its effect on insulin secretion; together these mechanisms prevent hypoglycemia. Incretins may also have a beneficial impact on beta cell mass and function, possibly decreasing the rate of beta cell apoptosis [21, 22]. GLP-1 and GIP are rapidly degraded by the enzyme dipeptidyl peptidase-4 (DPP-4), accounting for their short half-life in vivo.

Patients with type 2 diabetes have a blunted incretin response that can be overcome by supraphysiologic levels of GLP-1 but not GIP [23–25]. This has led to the discovery of the incretin mimetic peptide group: GLP-1 receptor agonists (GLP-1RAs) that act on the GLP-1 receptor but are resistant to degradation by DPP-4 [20]. They are administered subcutaneously and are available in shorter-acting daily or longer-acting weekly formulations. Longer-acting GLP-1RAs have a greater impact on fasting glucose, while shorter-acting GLP-1RAs have a greater effect on post-prandial glucoses [26]. GLP-1RAs approved for use in the United States include exenatide, liraglutide, and albiglutide [27]. DPP-4 inhibitors, also known as incretin enhancers, are oral medications that act by competitively and reversibly binding the active site of DPP-4 preventing the breakdown of endogenous GLP-1 and GIP [28]. There are several DPP-4 inhibitors currently on the market including sitagliptin, saxagliptin, linagliptin, and alogliptin [27].

### Considerations for use in the inpatient setting

Incretin-based therapies would be desirable in the inpatient setting for several reasons. Due to their glucose dependent actions, they carry a low risk of hypoglycemia, especially when used as monotherapy. By augmenting glucose-dependent insulin secretion and inhibiting glucagon secretion these medications also target the underlying pathophysiology found in patients with stress-induced hyperglycemia [13]. Dose reduction may be required for patients with renal insufficiency, but otherwise they require very little titration. Pharmacokinetics vary between the different agents, but in general the effect on glucose lowering can be seen relatively quickly, on the order of hours to days [28–30]. Finally, incretin-based therapies can be used in combination with insulin to provide further flexibility for blood glucose lowering than what can be obtained by incretins alone. These properties combined with a favorable short-term side effect profile make incretins attractive alternatives or adjuncts to insulin in the inpatient setting.

The most frequent reported side effects of GLP-1 and GLP-1RAs in the inpatient setting are gastrointestinal, including nausea, and vomiting [31]. These side effects could be particularly important with the initiation of therapy in the inpatient setting because they tend to be more common when starting therapy and decline with time. With regard to DPP-4 inhibitors, there was a small increase in infection risk including nasopharyngitis (6.4 vs 6.1 %, RR 1.2) and urinary tract infection (3.2 % vs 2.4 %, RR 1.5) [31]. Allergic reactions, although rare, have been reported [32]. While there was initially concern that incretin-based therapies may be associated with pancreatitis, multiple retrospective studies and pooled clinical trial data has failed to find such an association. In large prospective trials of two DPP-4 inhibitors saxagliptin and alogliptin, the risk of acute or chronic pancreatitis was not increased in diabetic patients followed up to 2 years when compared to placebo [33, 34]. Studies examining GLP1-RAs are still ongoing. Interestingly, baseline data from a cohort of patients in a study evaluating the GLP1-RA liraglutide found that lipase and amylase levels were above the upper limit of normal in 16.6 % and 12 % of diabetic patients respectively and often associated with renal dysfunction [35]. Studies that include longer follow-up and stringent clinical definitions to draw definitive conclusions about incretins and pancreatitis (especially GLP1RAs) are ongoing. With regard to cardiac outcomes, an increased rate of hospitalization for congestive heart failure has been linked to saxagliptin [33], however, this has not been found in other incretin agents. Indeed, animal models have suggested a cardioprotective effect of incretins in both ischemic heart disease and heart failure that has not been seen yet in human trials [36]. Again, further studies are needed to clarify if these results translate to major cardiac events, and if so, what subset of patients is most likely to benefit.

### Incretins in the ICU setting

The safety and efficacy of GLP-1 and GLP-1RAs to treat hyperglycemia have been evaluated in small studies in multiple ICU populations including cardiac, medical, surgical and burn. Most of the early studies have evaluated the cardiovascular benefits of GLP-1 infusion in patients with recent myocardial infarction or heart failure. These studies have shown varying degree of improvement in overall clinical outcome in patients with acute myocardial infarction with decreased left ventricular ejection fraction (LVEF) and heart failure [37–40]. The focus of this review is the effect of incretins on glycemia only.

### Inpatient use of intravenous GLP-1 without nutrition

#### Cardiac ICU

An early nonrandomized pilot evaluated the safety and efficacy of a 72 h GLP-1 infusion in cardiac ICU patients (n = 21, 50 % with diabetes) with acute myocardial infarction and decreased LVEF after a successful primary angioplasty [37]. The primary outcome was post infusion LVEF, which improved in all patients in the GLP-1 group. As a minor observation, the authors noted that mean plasma glucose levels improved in the first 48 h of the infusion from 162 to 126 mg/dl, and were accompanied by a reduction in plasma insulin. When patients began eating, glucose levels rose only to baseline fasting levels (168 mg/dl) prior to the infusion, and were associated with an increase in mean insulin levels from 184 to 301 pmol/L (p < 0.05). Glucose responses were not evaluated separately in diabetes patients [37]. Transient nausea was the major side effect noted mostly in patients without diabetes. In another study of 12 patients with congestive heart failure (8 with diabetes), five weeks of GLP-1 infusion improved glycemic control compared to 9 controls (5 with diabetes) as evaluated by an improvement in Hba1c from average 7.7 ± 0.4 to 6.9 ± 0.4 % [38]. It is important to note that cardiac ICU studies have mainly evaluated LVEF as their primary outcome and that glucose has not been a major focus of these studies.

#### Cardiac surgery

Several studies in coronary artery bypass patients have evaluated GLP-1. In a study of twenty patients undergoing coronary artery bypass graft surgery (CABG), ten patients (2 with diabetes) were started on a GLP-1 infusion 12 h before surgery and were continued on it perioperatively at 1.5pmol/kg/min for 48 h [39]. Compared to the control group, the GLP-1 group had better preoperative and intra-operative glucose levels, comparable postoperative glucose levels, and required 45 % less insulin to achieve a target glucose of 140 mg/dl. The hemodynamic results were similar and arrhythmias were reduced in the GLP-1 group. Another study evaluated GLP-1 infusion at 3.6 pmol/kg/min for 12 h after CABG in patients with insulin naïve type 2 diabetes [40]. Glucose control was similar in the GLP-1 and insulin groups but insulin requirements and the frequency of insulin adjustments were significantly lower in the GLP-1 group, especially within the first 6 h.

In a recent study of patients with and without diabetes, intraoperative GLP-1 in addition to a standard insulin protocol achieved better glucose control in the GLP-1 group during the CABG period (p = 0.009) [41].

#### Post-surgical

Meier et al. evaluated the efficacy of GLP-1 in a trial involving 8 patients with type 2 diabetes treated with oral hypoglycemic agents undergoing major surgical procedures [42]. 1.2 pmol/kg/min of GLP-1 was infused for 8 h and compared to placebo infused during a separate 8-hour period. Plasma glucose was significantly lower and reached normal fasting range within 150 min of GLP-1 infusion compared to persistent hyperglycemia in the control period (p < 0.001).

A recent study compared 18 post-surgical patients (9 with diabetes) receiving either intravenous GLP-1 at 1.5 pmol/kg/min or normal saline along with an insulin infusion for glucose control. GLP-1 along with insulin reduced the glycemic variability compared to the saline group and there were no major side effects noted [43].

### Inpatient use of GLP-1 with nutrition

Deane et al. evaluated incretins for treatment of hyperglycemia associated with enteral nutrition in patients with and without diabetes [44, 45]. In 7 mechanically ventilated patients without diabetes GLP-1 infusion at 1.2 pmol/kg/min was compared to placebo given on a separate day for hyperglycemia caused by post-pyloric nutrition. Use of GLP-1 significantly attenuated the hyperglycemia with peak glucose of 180 mg/dl compared to 216 mg/dl in controls [44]. A similar study was performed in 11 mechanically ventilated diabetes patients on oral agents before admission. GLP-1 attenuated the glucose response to <180 mg/dl in over 50 % of patients versus 36 % of the controls, with mean glucose of 205 compared to 228 mg/dl in controls [45].

Nauck et al. studied 9 surgical ICU patients (4 with diabetes) on TPN and 8 healthy controls on intravenous glucose therapy [46]. GLP-1 was given at 1.2 pmol/kg/min for 4 hrs. vs placebo for 4 hrs. GLP-1 infusion significantly lowered glucose to normoglycemic levels in healthy control and attenuated hyperglycemia in patients on total parenteral nutrition (TPN) from peak glucose of 211 to 159 mg/dl; 7 out of 9 patients had blood glucose less than 150 mg/dl on GLP-1 infusion.

### Inpatient use of GLP-1RAs without nutrition

Several trials have evaluated GLP-RA therapy in the ICU settings. Abuannadi et al. compared 40 patients with hyperglycemia (30 with diabetes on oral agents or basal insulin before admission) who received exenatide infusion to historical controls on an insulin infusion protocol with target glucose of 90-119 mg/dl (intensive control era-INT) and 100–140 mg/dl (moderate control era-MOD) [47]. Steady state glucose of 132 mg/dl was reached within 2 h in the exenatide group compared to 12 h (mean glucose 127 mg/dl) in MOD and 3 h (mean glucose 105 mg/dl) in the INT group. Mecott et al. evaluated subcutaneous exenatide plus insulin therapy in 6 pediatric burn patients and compared them to 18 controls receiving intensive insulin therapy. Glucose values and variability were the same in the two groups, however patient receiving exenatide required significant less insulin compared to controls [48].

Marso et al. used intravenous exenatide therapy in cardiac ICU patients and compared it to historical controls on insulin therapy and found significantly decreased time to steady state glucose. With exenatide therapy, a glucose level of 137 mg/dl was reached within 3.9 h compared to 9.3 h in the recent control group [49]. In another study conducted on patients undergoing elective CABG, Haluzik et al. evaluated intravenous exenatide along with insulin. Adding exenatide improved glucose control without increasing the incidence of hypoglycemia in these patients [50].

Summarizing the ICU trials, incretin monotherapy was most effective in stress hyperglycemia or parenteral and enteral feeding-induced hyperglycemia in patients without diabetes. In diabetic patients on oral agents with presumably mild to moderate insulin resistance, glucose control was successful with incretins alone, but insulin was required in those with significant insulin resistance. Here incretin use decreased the cumulative insulin dose, the frequency of insulin titrations, the time to reach steady state glucose and glucose variability. Several of these improvements could potentially improve workflow and outcomes in hospitalized patients**.** It is important to remember, however, that patients with diabetes on large insulin doses were excluded from these studies.

The most common side effects of GLP-1 in the ICU were nausea and vomiting, occurring in more than 50 % of the patients using doses of 1.5 pmol/kg/min or higher. [37, 38]. In newer studies using a lower dose GLP-1 at 1.2 pmol/kg/min, gastrointestinal side effect were lower. With exenatide therapy 15–20 % patients had gastrointestinal side effects [47, 49]. Incretin therapy is known to slow gastric emptying and may increase the risk of aspiration especially in the mechanically ventilated ICU patients. Hence, Deane et al. investigated effect on gastric emptying on 25 mechanically ventilated patients in the medical ICU. Patients receiving GLP-1 infusion at 1.2 pmol/kg/min with normal gastric emptying prior to therapy had significant slowing of their gastric emptying [51]. However, in patients with known reduced gastric emptying there was no further worsening. Interestingly, only patients with normal gastric emptying had significant improvement in their glycemia while patients with delayed gastric emptying at the start only showed a trend for reduction in postprandial glycemia.

Hypoglycemia is another major concern with the use of these agents. 1–2 events of hypoglycemia per 10 patients were recorded in the early ICU studies using 1.5 pmol/kg/min of GLP-1 but diminished with dose reduction. Studies using 1.2 pmol/kg/min did not have any severe hypoglycemic events [37, 39]. Although exenatide infusion was associated with varied frequency of hypoglycemia (0.1–10 %), this was significantly lower than with insulin [41, 47, 49, 50].

### Incretins in the general wards setting

There is currently little data on the use of incretins in the inpatient general ward setting. An open-label pilot study performed by Umpierrez et al. examined the safety and efficacy of sitagliptin in general medicine and surgery floor patients [52]. Strict criteria were used to include diabetic patients treated with diet, oral agents, or insulin at a total daily dose of less than 0.4 units/kg/day. Patients were randomized to sitagliptin once daily, sitagliptin with basal insulin, or a traditional basal-bolus regimen. All groups received correctional scale insulin. Glycemic control was similar between the groups with no difference in hypoglycemia or length of hospitalization between the two groups. The sitagliptin groups required a lower total daily dose of insulin and fewer insulin injections compared to those in the traditional basal-bolus group. Glucose at randomization was a predictor of glucose control and patients with blood glucose < 180 mg/dl at randomization had improved blood glucose profile with treatment.

A larger randomized multicenter trial comparing sitagliptin with or without basal insulin to a traditional basal-bolus regimen is currently ongoing [53]. Further data is needed to determine the role for incretin-based therapies in the inpatient setting

### Future directions

Schwartz and Defronzo have outlined a process of care to use incretins on admission and until discharge for patients with stress hyperglycemia, prediabetes and diabetes, both on the floor and in the ICU [54]. They suggest starting these agents prior to, or immediately on an elective admission, and note this prevents hyperglycemia in majority of diabetes patients. In the case of persistent hyperglycemia, insulin was added, initially as a correction scale and then migrated to basal insulin if necessary. They also suggest that incretin based therapies be continued until discharge, when patients could be transitioned back to their home diabetes regimens. There are no large studies validating this approach.

## Conclusions

Hyperglycemia management using the current gold standard of therapy, insulin, improves morbidity in hospitalized patients. Insulin treatment, although widely used, is labor-intensive in nature and carries a significant risk of hypoglycemia. Incretin-based medications as alternative or additive therapies to insulin in the hospital setting have shown potential in small pilot ICU and general ward studies of glucose control. Larger studies using incretin-based therapies are necessary to establish their safety and efficacy in the hospital setting.

## Abbreviations

CABG: Coronary artery bypass graft surgery
DPP-4: Dipeptidyl peptidase-4
GLP-1RA: Glucagon-like peptide-1 receptor agonists
GIP: Glucose-dependent insulinotropic polypeptide
GLP-1: Glucagon-like peptide-1
ICU: Intensive care unit
INT: Intensive control era
LVEF: Left ventricular ejection fraction
MOD: Moderate control era
TPN: Total Parenteral Nutrition
